# Oral Administration of *Faecalibacterium prausnitzii* Decreased the Incidence of Severe Diarrhea and Related Mortality Rate and Increased Weight Gain in Preweaned Dairy Heifers

**DOI:** 10.1371/journal.pone.0145485

**Published:** 2015-12-28

**Authors:** Carla Foditsch, Richard Van Vleck Pereira, Erika Korzune Ganda, Marilia Souza Gomez, Eduardo Carvalho Marques, Thiago Santin, Rodrigo Carvalho Bicalho

**Affiliations:** 1 Department of Population Medicine and Diagnostic Sciences, College of Veterinary Medicine, Cornell University, Ithaca, New York, United States of America; 2 Department of Clinical Science, College of Veterinary Medicine and Animal Sciences, University of São Paulo, São Paulo—SP, Brazil; GI Lab, UNITED STATES

## Abstract

Probiotics are a promising alternative to improve food animal productivity and health. However, scientific evidence that specific microbes can be used to benefit animal health and performance is limited. The objective of this study was to evaluate the effects of administering a live culture of *Faecalibacterium prausnitzii* to newborn dairy calves on subsequent growth, health, and fecal microbiome. Initially, a safety trial was conducted using 30 newborn bull calves to assess potential adverse effects of the oral and rectal administration of *F*. *prausnitzii* to neonatal calves. No adverse reactions, such as increased body temperature or heart and respiratory rates, were observed after the administration of the treatments. All calves survived the experimental period, and there was no difference in fecal consistency score, attitude, appetite or dehydration between the treatment groups. The rectal route was not an efficient practice while the oral route ensures that the full dose is administered to the treated calves. Subsequently, a randomized field trial was completed in a commercial farm with preweaned calves. A total of 554 Holstein heifers were assigned to one of two treatment groups: treated calves (FPTRT) and non-treated calves (control). Treated calves received two oral doses of *F*. *prausnitzii*, one at treatment assignment (1^st^ week) and another one week later. The FPTRT group presented significantly lower incidence of severe diarrhea (3.1%) compared with the control group (6.8%). Treated calves also had lower mortality rate associated with severe diarrhea (1.5%) compared to control calves (4.4%). Furthermore, FPTRT calves gained significantly more weight, 4.4 kg over the preweaning period, than controls calves. The relative abundance of *F*. *prausnitzii* in the fecal microbiota was significantly higher in the 3^rd^ and 5^th^ weeks of life of FPTRT calves than of the control calves, as revealed by sequencing of the 16S rRNA gene. Our findings showed that oral administration of *F*. *prausnitzii* improves gastrointestinal health and growth of preweaned calves, supporting its use as a potential probiotic.

## Introduction

Diarrhea is one of the major causes of morbidity in preweaned dairy heifers, resulting in significant economic losses and animal suffering. The U.S. Department of Agriculture (USDA) surveyed morbidity and mortality rates in preweaned heifers from heifer-raising operations in 2006 [1]. Digestive disorders (i.e. diarrhea and bloat) affected 23.9% of the population; the overall mortality rate was 7.8% and 56.5% of the deaths were caused by digestive problems, mainly diarrhea [1]. The high incidence of diarrhea is a persistent problem. Four years after the 2006 survey, the USDA reported that 25.3% of preweaned calves had diarrhea; the overall mortality rate decreased to 4.2% and digestive problems were responsible for one third of the deaths. [2]. The treatment of diarrhea with antibiotics, supportive care and labor is expensive, thus development of new strategies to prevent diarrhea will maximize overall productivity, animal welfare and profitability.

Historically, sub-therapeutic doses of antibiotics have been used as growth promoters and disease prophylaxis in livestock animals. The World Health Organization global report on surveillance of antimicrobial resistance cited *Escherichia coli*, *Klebsiella pneumoniae and Staphylococcus aureus* as examples of bacteria that exhibit high rates of resistance in all regions surveilled [3]. Due to the emergence of antibiotic resistant microbes, the use of antibiotics as growth promoters is now banned in the European Union and is limited in the United States. Effective in the spring of 2015, the Food and Drug Administration published new directives for the use of antimicrobials in the feed of livestock in the United States, creating a new category of products called veterinary feed directive drugs (VFD drugs). Under this directive, a VFD drug intended for use in or on animal feed must be used under the professional supervision of a licensed veterinarian [4]. The use of probiotics is a potential alternative to the use of antimicrobials in livestock feed.

*Faecalibacterium prausnitzii* belongs to the phylum *Firmicutes* and is an obligate anaerobic, Gram-positive, rod-shaped, butyrate producing microorganism [5,6] that is abundant in the feces of several animal species [7–13]. In humans, high levels of *F*. *prausnitzii* were associated with obesity [14], while a low abundance of *F*. *prausnitzii* was linked to Inflammatory Bowel Disease (IBD, i.e. Crohn’s disease [15,16] and ulcerative colitis [17]). *F*. *prausnitzii* has anti-inflammatory properties, which have been demonstrated *in vitro* with cultured cells and *in vivo* with trinitrobenzenesulfonic acid (TNBS)-induced colitis in mice models [16,18–20]. *F*. *prausnitzii* induces the production of the anti-inflammatory cytokine IL-10 and reduces the secretion of the pro-inflammatory cytokines IFN-γ and IL-12 [20]. Furthermore, *F*. *prausnitzii* and its supernatant decreased the severity of colitis in IBD mice models [16,18]. Additionally, the butyrate produced by *F*. *prausnitzii* is both an energy source to enterocytes and act as an anti-inflammatory agent [21].

In preweaned Holstein calves, higher relative abundance of *F*. *prausnitzii* in the first week of life was associated with enhanced weight gain and reduced incidence of diarrhea [10]. A recent study conducted by our research group isolated 203 *F*. *prausnitzii* isolates from the feces of calves and piglets [5]. In that study, 40 genetically distinct *F*. *prausnitzii* isolates were selected for further characterization. A large variability was observed among isolates for *in vitro* short chain fatty acids (SCFA) metabolism, growth, antibiotic resistance, and sensitivity to low pH and bile salts. Based on this data, 4 isolates with desirable characteristics were selected and used as part of a probiotic cocktail in the *in vivo* studies described herein.

The first objective of the present study was to assess the safety of the oral and rectal administration of a live culture of *F*. *prausnitzii*. The second objective was to evaluate the effects of the oral administration of *F*. *prausnitzii* on preweaned calves’ survivability, incidence of severe diarrhea, weight gain and fecal microbiome.

## Materials and Methods

### Ethics statement

This study was carried out in strict accordance with the recommendations of The Animal Welfare Act of 1966 (AWA) (P.L. 89–544) and its amendments 1970 (P.L. 91–579); 1976 (P.L. 94–279), and 1985 (P.L. 99–198) which regulate transport, purchase, care, and treatment of animals used in research. The research protocol was reviewed and approved by the Institutional Animal Care and use Committee of Cornell University (Protocol number: 2012–0055). The administration of *F*. *prausnitzii* culture to calves housed on the commercial dairy farm was authorized by the farm owner, who was aware of all experimental procedures.

### Treatment preparation

Four *F*. *prausnitzii* isolates were selected from our culture collection based on greater capacity for *in vitro* butyrate production, growth and tolerance to low pH and bile salts as previously evaluated by our research group [5]. The four isolates (ref. numbers 34, 35, 1S, and 2S; Foditsch et al. (2014)) were cultured individually in a medium supplemented with 30% ruminal fluid as previously described [5]. The average colony forming units (CFU) of each isolate was 1.43 x 10^7^ CFU/mL. Equal volumes of the four cultures were mixed, frozen in 50 mL sterile disposable centrifuge tubes with 15% glycerol, and stored at -80°C. For quality assurance purposes, the CFU/mL was calculated at the time of administration; the average CFU was 1.34 x 10^7^ CFU/mL, confirming that a live bacteria culture was administered to the calves. The placebo given to control calves in the safety trial contained the same growth medium without the bacterial culture.

### Safety trial

#### Animals and facilities

The safety trial was conducted from March to May of 2014 at the College of Veterinary Medicine, Cornell University. Thirty bull calves were obtained from a commercial dairy farm that milked 2,800 Holstein cows near Ithaca, New York, USA. Immediately after birth, calves were removed from the maternity pens and were placed in dry sawdust bedded pens. Four liters of pooled, non-pasteurized colostrum from primiparous cows was administered to calves by esophageal feeder. The primary researcher, which is a veterinarian, used an authorized van with individual transportation cages to transport the newborn calves to the College of Veterinary Medicine facility, where they were housed individually in 2.2 x 1.5 meters concrete stalls bedded with pine shavings. Calves were kept in the same stall during the 14 days of the research trial. Non-pasteurized whole milk was fed twice daily at approximately 10% of the body weight and water was available *ad libitum*. Stalls were kept clean and environmental enrichment utensils were used to minimize animal stress. No animal suffering was anticipated as a result of the trial, therefore analgesics and anesthetics were not administered. All animals were sold alive after the trial.

#### Study design and data collection

A randomized clinical trial design was used. Thirty calves were randomly allocated into one of four treatment groups as follows: oral control (n = 5) calves received 80 mL of a placebo solution orally; oral treatment (n = 10) calves received 80 mL of live culture of *F*. *prausnitzii* orally; rectal control (n = 5) calves received 80 mL a placebo solution rectally; and rectal treatment (n = 10) calves received 80 mL of live culture of *F*. *prausnitzii* rectally. Control groups received a placebo containing the growth medium without the bacterial culture. Oral treatments were administered through an esophageal tube and rectal treatments were given with a 6 cm drench tube attached to a syringe. Treatments were administered on the second day of life in order to avoid interactions between colostrum’s immune cells and the bacteria administered. Due to the *F*. *prausnitzii* sensitivity to low pH [5], the treatments were administered 1 hour after milk feeding, when the abomasal pH increases approximately from 2 to 6 [22]. Calf health was assessed twice daily by the primary researcher for the following parameters; fecal consistency (0 = well-formed; 1 = semi-formed; 2 = loose or watery feces not containing blood; and 3 = loose or watery feces containing blood), dehydration (0 = euhydrated; 1 = skin tented 2 to 6s; 2 = skin tented 6 to 10s; and 3 = skin tented ≥ 10s), attitude (0 = alert; 1 = depressed; and 2 = non responsive) and appetite (0 = normal; 1 = consumed ½ bottle; 2 = consumed 1/4 bottle; and 3 = forced fed). The effect of treatment on fecal consistency, dehydration, attitude and appetite scores was assessed using ordinal logistic regression models fitted in JMP Pro 11 (SAS Institute Inc., NC, USA). The independent variables offered to the model were treatment group, age in days, and interaction terms between treatment and age.

### Randomized field trial

#### Farm and management

The study was conducted from November 2014 to April 2015 at a commercial dairy farm. Immediately after birth, female calves were removed from the maternity pens, weighed, and placed in dry sawdust bedded pens. Four liters of pooled non-pasteurized colostrum from primiparous cows was administered to calves by esophageal tubing and calves had their umbilicus dipped in 7% iodine solution.

Newborn calves were transported twice daily from the maternity area to the calf barn. Calves were housed in a green-house barn divided into 30 identical pens with positive ventilation. Pens were separated by steel gates and calves were moved by birth order into each pen until maximum capacity was reached (20 calves/pen). Calves remained in the same pen until weaning.

Calves were fed *ad libitum* acidified non-saleable milk using a fully automated system with 6 nipples per pen. Acidification was performed in a sealed stainless-steel tank where cold milk (5°C) was mixed with organic acid under constant homogenization until a pH of 4.5 was reached. Acidified milk was directed to a smaller stainless-steel tank, warmed, and distributed to the pens. Acidified milk was offered to the calves from day one to 56 of life. All calves were weaned by reducing the daily milk availability starting on day 42 until complete absence of acidified milk at 57 days of life. Water and solid feed (calf starter mix) were offered *ad libitum* to all calves.

Health-related events (e.g. otitis, pneumonia and severe diarrhea) were recorded and treated as needed by farm employees. One dose of the macrolide antibiotic Zuprevo (Merck Animal Health, Summit, NJ) was given by the farm to all female calves at eight to 14 days of age as a metaphylactic for bovine respiratory disease. All calves were disbudded by heat cauterization at approximately four weeks of age.

#### Study design and data collection

The treatment administered was a live microorganism and cross-contamination between calves in the same group was possible. Therefore, all calves in the same pen were assigned to the same treatment group (oral treatment with *F*. *prausnitzii* (FPTRT) or control, at 5 ± 2 days of life). The first group was randomly selected, and the subsequent groups were alternated between control and FPTRT, resulting in the same number of calves for each treatment group per week.

The rumen microbiota gradually changes from aerobic to anaerobic during the calves’ first weeks of life [23–25], therefore we chose to treat calves in the field trial with two 40 ml doses of *F*. *prausnitzii* culture, one dose at treatment assignment (1^st^ week of life) and a second dose one week later, instead of only administering one 80 ml dose on the second day of life, to increase the chances of its colonization in the large intestine. The control calves did not receive a placebo treatment or sodium bicarbonate.

In a group feeding system it was not possible to determine the time each calf was fed and to account for the increase of the abomasal pH, as in the safety trial. Additionally, the milk fed in the commercial farm was acidified and, as mentioned previously, *F*. *prausnitzii* is highly sensitive to low pHs. Therefore, we administered 130 mL of sodium bicarbonate (90 mg/mL) orally to FPTRT calves to buffer the low pH of the abomasum before administering the culture. Sodium bicarbonate at 0.6% was used previously to increase the pH of fermented waste milk to 6.0 in a study evaluating feeding value of fermented waste milk [26]. In that study, calves received one of the four milk treatments (fresh milk, fresh waste milk, fermented waste milk or fermented waste milk with sodium bicarbonate) for 42 days and weight gain was not significantly different between groups. We estimated the dose of sodium bicarbonate considering the milk present in the abomasum and did not expect effects of the two administrations of bicarbonate, other than the neutralization of the abomasal pH prior to *F*. *prausnitzii* administration.

A total of 554 Holstein heifers were enrolled in the field trial, with 296 allocated to the control group and 258 to the FPTRT group. A subset of 35 calves/treatment was selected randomly for collection of fecal DNA through rectal swabs and evaluation of fecal microbiome. From these 70 calves, 45 calves (n = 22, control; n = 23, FPTRT) were selected randomly for evaluation of serum β-hydroxybutyrate (BHBA) concentrations. Blood samples were collected from the jugular vein and fecal samples were collected using rectal swabs on the 1^st^ (enrollment), 3^rd^, 5^th^ and 7^th^ weeks of life. Blood samples were centrifuged at 3000 x g for 10 minutes, after which serum was obtained. Serum and swabs were stored at -20°C until assayed. Fecal consistency scores were recorded weekly using a four level scoring system, as described in the safety trial. Calves were weighed using a Waypig 15 digital scale (Vittetoe Inc., Keota, IA, USA) at birth and again at weaning (56 ± 3 days of life; n = 141 for the control group and n = 146 for FPTRT group). Weight gain was calculated by subtracting the birth weight from the weight at weaning. The weight gain was divided by the age in days at the second weight (56 ± 3 days) to obtain the average daily gain (ADG). Due to equipment constraints, weights of a subset of calves (303) were obtained. Severe diarrhea and death events records were acquired from the farm’s software (Dairy-Comp 305; Valley Ag Software, Tulare, CA, USA). Severe diarrhea was defined as dehydrated calves with loose or watery feces that were treated by the farm employees with oral electrolytes or intravenous fluids. Farm employees were blind to the treatment groups.

The treatment performed was not expected to be associated with decreased health and performance. Animals housed on commercial dairy farms may become ill for a variety of reasons and ensuing mortality is inevitable. When animals are diagnosed with a disease, treatment is attempted and, if animals are too sick to respond to treatment, euthanasia may be performed. As researchers, performing research with privately owned animals, we cannot interfere with farm protocols. Additionally, we do not have information if the animal died with or without euthanasia since our researcher was not involved in this decision and action. Nevertheless, our IACUC protocol described in details the circumstances of our proposed study and that protocol was thoroughly reviewed and approved by the committee.

#### DNA extraction, amplification and purification

DNA of the fecal material from the four time points (1^st^, 3^rd^, 5^th^ and 7^th^ week of life) was extracted following the protocol previously used by Oikonomou et al. (2013). Briefly, each rectal swab was placed in 1.5 ml of nuclease-free water (Life Technologies, Grand Island, NY) and vortexed for at least two minutes. The swab was then removed and the sample centrifuged for 10 min at 13,200 x g. The supernatant was discarded and the remaining pellet was resuspended in 400 μl of nuclease-free water. Isolation of microbial genomic DNA was performed by using a QIAamp DNA minikit (Qiagen, Germantown, MD) according to the manufacturer’s instructions. Besides the proteinase K and the Buffer AL, 40 μl (10 mg/ml) of lysozyme (Sigma-Aldrich, St. Louis, MO) were added to the sample and the incubation at 56°C was extended for 12 h. The DNA concentration and purity were evaluated by optical density using a NanoDrop ND-1000 spectrophotometer (NanoDrop Technologies, Rockland, DE, USA) at wavelengths of 230, 260 and 280 nm.

The 16S rRNA gene was amplified by PCR from individual metagenomic DNA samples using barcoded primers. For amplification of the V4 hypervariable region of the bacterial/archaeal 16S rRNA gene, primers 515F and 806R were used according to a previously described method optimized for the Illumina MiSeq platform (Illumina, Inc., San Diego, CA, USA) [27]. The earth microbiome project [28] was used to select 280 different 12-bp error-correcting Golay barcodes for the 16S rRNA PCR, as previously described [27]. The 5'-barcoded amplicons were generated in triplicate using 1μL DNA template, 2 X EconoTaq® Plus Green Master Mix (Lucigen®, Middleton, WI, USA), and 5 μM of each primer. The PCR conditions for the 16S rRNA gene consisted of an initial denaturing step of 94°C for 3 min, followed by 35 cycles of 94°C for 45 s, 50°C for 1 min, and 72°C for 90 s, and a final elongation step of 72°C for 10 min. Blank controls, in which no DNA was added to the reaction, were performed for quality assurance. Replicate amplicons were pooled and visualized by electrophoresis through 1.2% (wt/vol) agarose gels stained with 0.5 mg/mL ethidium bromide. Amplicons were purified with a PCR DNA extraction kit (IBI Scientific, Peosta, IA, USA) and the purified 16S rRNA amplicons were quantified using the Qubit dsDNA BR assay kit (Life Technologies, Carlsbad, CA, USA) and a Qubit fluorometer (Life Technologies).

#### Sequence library analysis and statistical analysis

Amplicon DNA aliquots were standardized to the same concentration and then pooled. Final equimolar libraries were sequenced using the MiSeq reagent kit v2 (300 cycles) on the Illumina MiSeq platform. The obtained 16S rRNA gene sequences were processed using the open source software pipeline Quantitative Insights Into Microbial Ecology (QIIME) version 1.7.0-dev [29]. Sequences were filtered for quality using established guidelines [30]. Sequences were binned into Operational Taxonomic Units (OTU) based on 97% identity using UCLUST [31] against the Greengenes reference database [32], May 2013 release. Low-abundance clusters were filtered and chimeric sequences were removed using USEARCH [31]. All samples were rarefied to an equal depth of 10,000 sequences using QIMME. The classification of reads at multiple taxonomic levels (kingdom, phylum, class, order, family, and genus) used in the present study were obtained from the MiSeq Reporter and are based on the Greengenes database cited above.

Using the obtained OTU information, we evaluated each sample’s richness using the Chao1 index, which is a nonparametric estimator of the minimum richness (number of OTU) and is based on the number of rare OTU (singletons and doublets) within samples. Microbiota diversity was measured using the Shannon index, which is a nonparametric diversity index that combines estimates of richness (the total number of OTU) and evenness (the relative abundance of OTU).

#### β-hydroxybutyrate analysis

β-hydroxybutyrate was measured for 180 serum samples. The Autokit Total Ketone Bodies (Wako Pure Chemical Industries Ltd., Richmond, VA, USA), a cyclic enzymatic method based on the oxidation of BHBA to acetoacetate by BHBA dehydrogenase, was chosen to measure serum BHBA due to its high sensitivity and high specificity.

### Statistical analysis

Pearson chi-square test was used to compare the following categorical variables between treatment groups: parity of the dam (1, 2, 3), occurrence of twins (yes or no), and calving ease of the dam (assisted or non-assisted).

Kaplan-Meier survival analysis were performed using MedCalc Statistical Software version 13.1.2 (MedCalc Software, Ostend, Belgium) to compare the effect of oral *F*. *prausnitzii* administration on the incidence of severe diarrhea cases, on the mortality rate caused by severe diarrhea and on the overall mortality rate.

The effect of oral administration of *F*. *prausnitzii* on fecal consistency score was assessed using an ordinal logistic regression model fitted in JMP. The independent variables offered to the model were treatment group (control, FPTRT), week of life (1–8), and interaction term between treatment and week.

The effects of oral administration of *F*. *prausnitzii* on weight gain and ADG were evaluated by linear regression models fitted in JMP with calf as the experimental unit. Variables offered to the models included treatment (control and FPTRT), birth weight, age at enrollment, age at weaning, parity of the dam (1, 2, 3), occurrence of twins, and calving ease of the dam (assisted or non-assisted). The interaction terms between treatment groups and all independent variables were evaluated in the model. Pen was fitted as a random effect. Manual backward variable elimination was undertaken considering main effects and interactions, which were retained in the model when *P* ≤ 0.05.

Additionally, the relative abundance of *F*. *prausnitzii* in the 1^st^ week of life of the subset of 70 calves was dichotomized in LowFP and HighFP. The mean relative abundance of *F*. *prausnitzii* and 95% confidence intervals were 0.42% (0.30–0.54) for the LowFP calves (n = 20 control, n = 18 FPTRT) and 17.99% (12.99–23.00) for the HighFP calves (n = 15 control, n = 17 FPTRT). ANOVA was used to evaluate the effect of the low and high abundance of *F*. *prausnitzii* in the first week of life on the weight gain of this subset of calves.

*Faecalibacterium*, *Firmicutes* and *Bacteroidetes* mean relative abundances, *Firmicutes* to *Bacteroidetes* ratio, and BHBA concentration were each compared using multiple linear mixed regression models in JMP. Variables offered to the models included treatment group, week of life, and the interaction terms between these two variables. Calf and pen were fitted as random effects. Number of OTU, Chao1 and Shannon indexes means were estimated using a similar linear mixed regression model described above.

## Results

### Safety trial

No adverse reactions, such as increased body temperature, heart and respiratory rates, were observed after the administration of the treatments and during the following days. All 30 bull calves survived the experimental period and there was no difference in fecal consistency score, attitude, appetite or dehydration between the four treatment groups (*P* ≥ 0.05. Supporting information S1, S2, S3 and S4 Figs). We concluded that it was safe to administer *F*. *prausnitzii* culture to newborn calves. Although the rectal administration was a promising way of by-passing the low pH of the abomasum and the detrimental effect of bile salts, it was not an efficient practice. Most of the infused liquid was promptly excreted by the calf. Therefore, the oral route was selected for the field trial.

### Randomized field trial

A total of 554 Holstein heifers were enrolled in this randomized field trial, 296 in the control group and 258 in the FPTRT group. A total of 22 were twins, 12 in the control group (4.10%) and 10 in the FPTRT group (3.89%; *P =* 0.99). Six control calves (2.05%) and seven FPTRT calves (2.72%) were born with assistance (*P =* 0.60). The numbers of calves born from first lactation cows were 166 for control calves (56.66%) and 124 (48.25%) for FPTRT calves; from second lactation cows were 70 in the control group (23.89%) and 72 in the FPTRT (28.02%), and from third or more lactations cows were 57 in the control group (19.45%) and 61 in the FPTRT (23.74%), *P =* 0.14).

**Fig 1:**
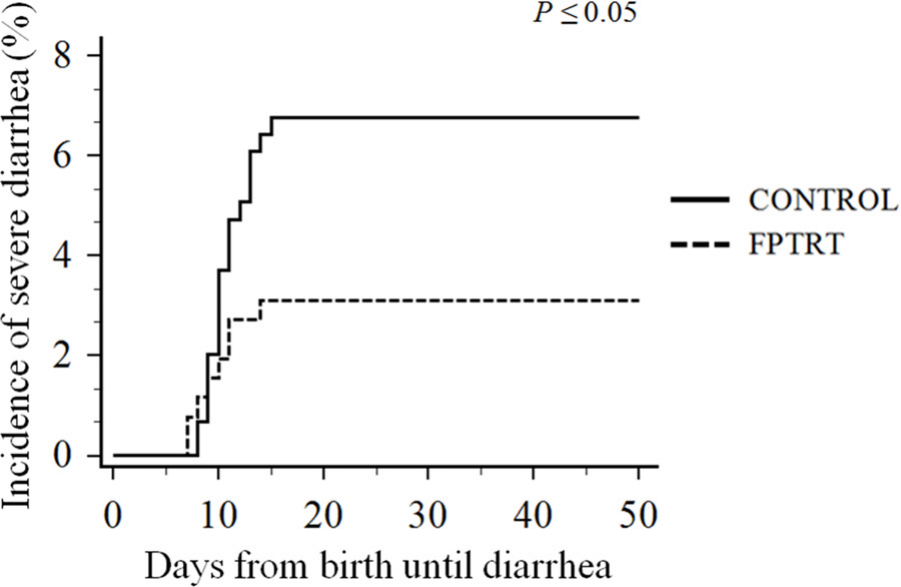
Incidence of severe diarrhea cases from birth to weaning of dairy calves. Field trial. Effect of *F*. *prausnitzii* administration on incidence of severe diarrhea according by treatment groups: FPTRT (N = 258) or control (N = 296). Severe diarrhea was defined as dehydrated calves with loose or watery feces that were treated by the farm employees with oral electrolytes or intravenous fluids.

**Fig 2:**
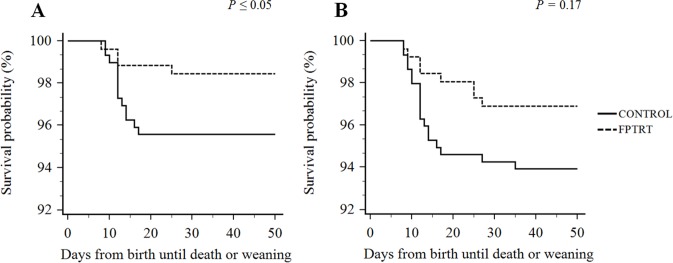
Effect of *F*. *prausnitzii* administration on mortality rate of preweaned dairy calves. Field trial. Kaplan-Meier survival analysis from birth to weaning of dairy calves by treatment groups: FPTRT (N = 258) or control (N = 296). A) Effect of *F*. *prausnitzii* administration on mortality rate related to severe diarrhea. B) Effect of *F*. *prausnitzii* administration on overall mortality.

Calves that were treated with *F*. *prausnitzii* had significantly lower incidence of severe diarrhea over the preweaning period compared to the controls, 3.1% and 6.8%, respectively (*P =* 0.05), as depicted in Fig 1. Mortality rate associated with severe diarrhea was also significantly lower for FPTRT calves, 1.5%, compared to control calves, 4.4% (*P =* 0.05), and the overall mortality was numerically lower for the FPTRT group compared to the control, 3.9% and 6.1%, respectively (*P =* 0.17). Survival analysis graphs are presented in Fig 2.

**Fig 3:**
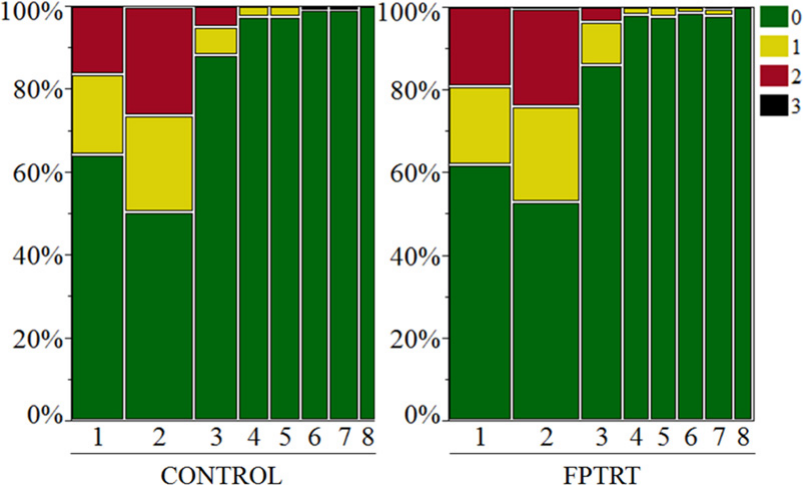
Mean fecal consistency score (0–3) during the preweaning period. Field trial. Dairy calves’ fecal consistency score distribution during the eight weeks of life by treatment groups: control and FPTRT. (0 = well-formed fecal samples; 1 = semi-formed fecal samples; 2 = loose or watery feces not containing blood; and 3 = loose or watery feces containing blood).

The fecal consistency scores did not differ between treatments (*P =* 0.38). Fig 3 describes the percentage of each fecal consistency score according to study group during the eight weeks of life of the treatment calves. Week of life had a significant effect on fecal consistency score (*P <* 0.0001). A higher incidence of diarrhea (score 2 and 3) was observed during the first two weeks of life.

Calves in the FPTRT group gained significantly more weight than calves in the control group during the preweaning period (*P* < 0.05). Birth weight, weaning weight, weight gained during the preweaning period and ADG values are presented in Table 1A. When comparing calves with high or low prevalence of *F*. *prausnitzii* in the 1^st^ week of life, the treatment had a significant effect on weight gain when administered to HighFP calves (*P* < 0.05), as shown in Table 1B.

**Table 1:** Weight gained and average daily gain during the preweaning period. Field trial.

**A**		Control		FPTRT		
		Mean	SE	Mean	SE	*P-*value
Birth weight, kg		36.0	0.32	36.8	0.27	0.08
Weaning weight, kg		75.9	1.08	80.2	1.05	< 0.01
Weight gain, kg		38.4	1.09	42.8	1.06	0.02
ADG, kg		0.68	0.02	0.77	0.02	0.01
**B**		Control		FPTRT		
		Mean	SE	Mean	SE	*P-*value
Weight gain, kg	LowFP	36.5	2.28	40.3	2.41	0.27
	HighFP	39.3	2.71	48.2	2.62	0.03
ADG, kg	LowFP	0.65	0.04	0.72	0.04	0.18
	HighFP	0.70	0.05	0.85	0.04	0.03

A) Effect of oral administration of *F*. *prausnitzii* to newborn dairy calves weight gain during the preweaning period (N = 303).

B) Effect of oral administration of *F*. *prausnitzii* to newborn dairy calves with high or low relative abundance of *F*. *prausnitzii* in the 1^st^ week of life (LowFP and HighFP; N = 70).

**Fig 4:**
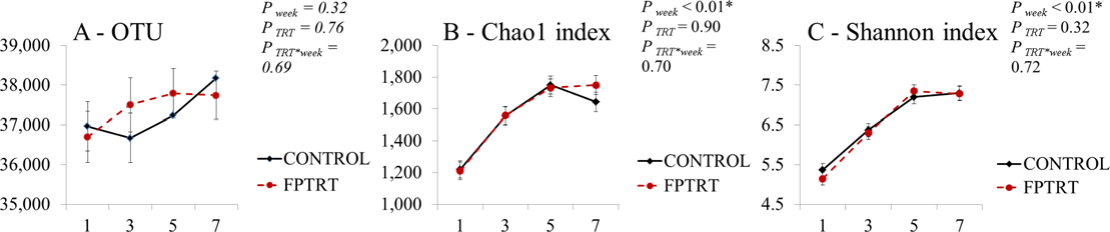
Operational taxonomic unit (OTU), Chao1 index and Shannon index. Field trial. OTU (A), Chao1 index (B) and Shannon index (C) according to treatment group. The error bars represent standard errors of the means.

From the 280 rectal swabs collected, DNA was successfully extracted from 264 samples. Quality-filtered reads for 16S sequences yielded a total of 16,266,816 sequences with an average coverage of 61,617 sequences per sample. The mean number of sequences per sample and the 95% confidence interval were: 62,218 (60,209–64,227) for the control group’s samples and 61,033 (59,055–63,012) for the FPTRT group’s samples. The effect of the interactions between the treatment groups and the week of life on the OTU, Chao 1 richness index and the Shannon diversity index are presented in Fig 4. There was a significant effect of week of life on the Chao 1 (*P* < 0.01) and Shannon (*P* < 0.01) indexes.

**Fig 5:**
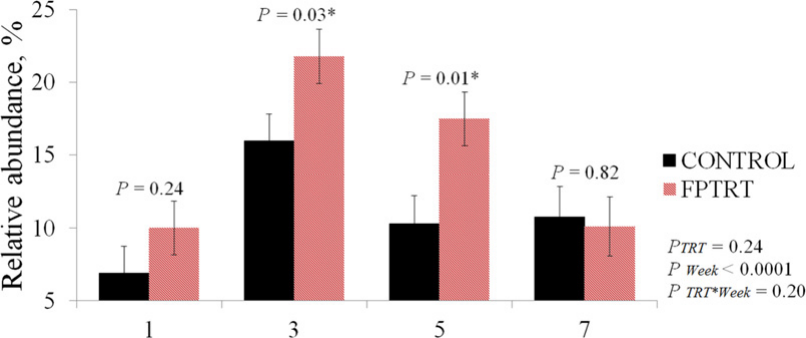
*Faecalibacterium* mean relative abundance. Field trial. *Faecalibacterium* mean relative abundance (Y axis, %) for each treatment group (control and FPTRT) over their 1^st^, 3^rd^, 5^th^ and 7^th^ week of life (X axis). The error bars represent the standard errors of the means.

**Fig 6:**
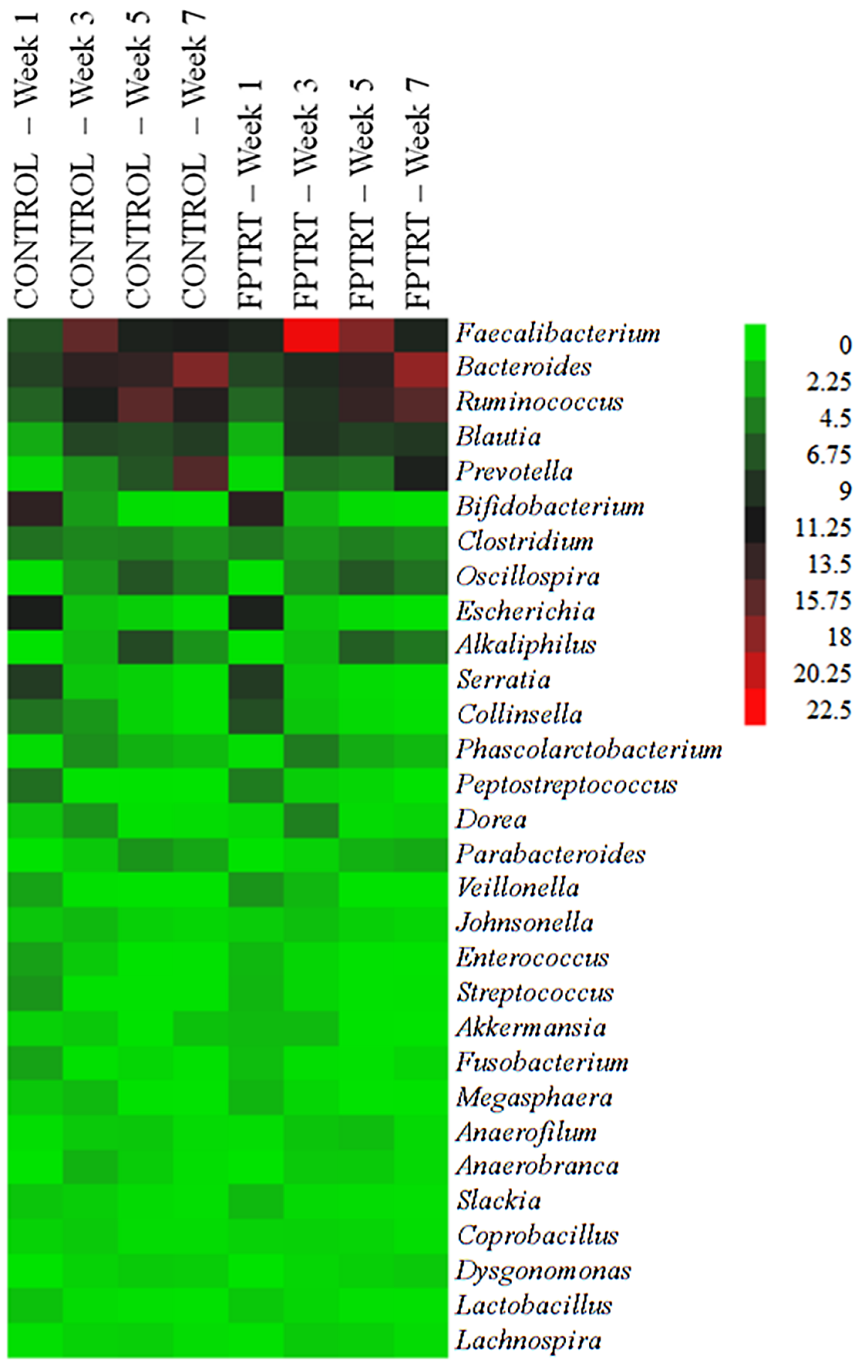
Heat map illustrating the relative abundance of the 30 most prevalent genera. **Field trial.** Heat map illustrating the relative abundance of the 30 most prevalent genera according to treatment group (control and FPTRT) over their 1^st^, 3^rd^, 5^th^ and 7^th^ week of life. The color and intensity of each square represent the value of the microbial relative abundance, as presented in the legend.

The mean relative abundance of the genus *Faecalibacterium* was significantly higher in the FPTRT group in the 3^rd^ and 5^th^ weeks of life (*P* < 0.05) compared to the control group, as illustrated in Fig 5. Other bacterial genera were not significantly different between the study groups. The 30 most common genera for the two treatment groups are shown in a heat map (Fig 6). *Faecalibacterium* (mean 13.0%), *Bacteroides* (mean 12.2%), *Ruminococcus* (mean 10.8%), *Blautia* (mean 6.5%), *and Prevotella* (mean 5.6%) were the five most prevalent genera during the preweaning period. *Escherichia* was the 9^th^ most prevalent genus (mean 3.3%), with an average prevalence of 10% in the first week of life and decreasing to less than 0.2% in the 7^th^ week.

**Fig 7:**
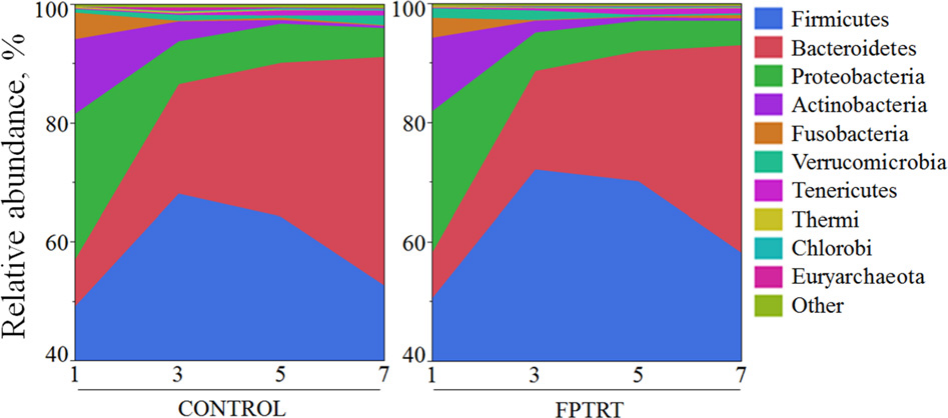
Aggregate microbiome composition at the phylum level. **Field trial.** Aggregate microbiome composition at the phylum level for 16S rRNA sequences according to treatment group (control and FPTRT) over their 1^st^, 3^rd^, 5^th^ and 7^th^ week of life. The y axis represents the mean relative abundance of OTUs for all samples evaluated within the specific week of life.

The relative abundance of the ten most prevalent phyla present in the fecal samples is illustrated by treatment group in Fig 7. *Firmicutes* was the most abundant phylum (mean 61.0%), followed by *Bacteroidetes* (mean 20.7%), and *Proteobacteria* (mean 10.6%).

**Fig 8:**
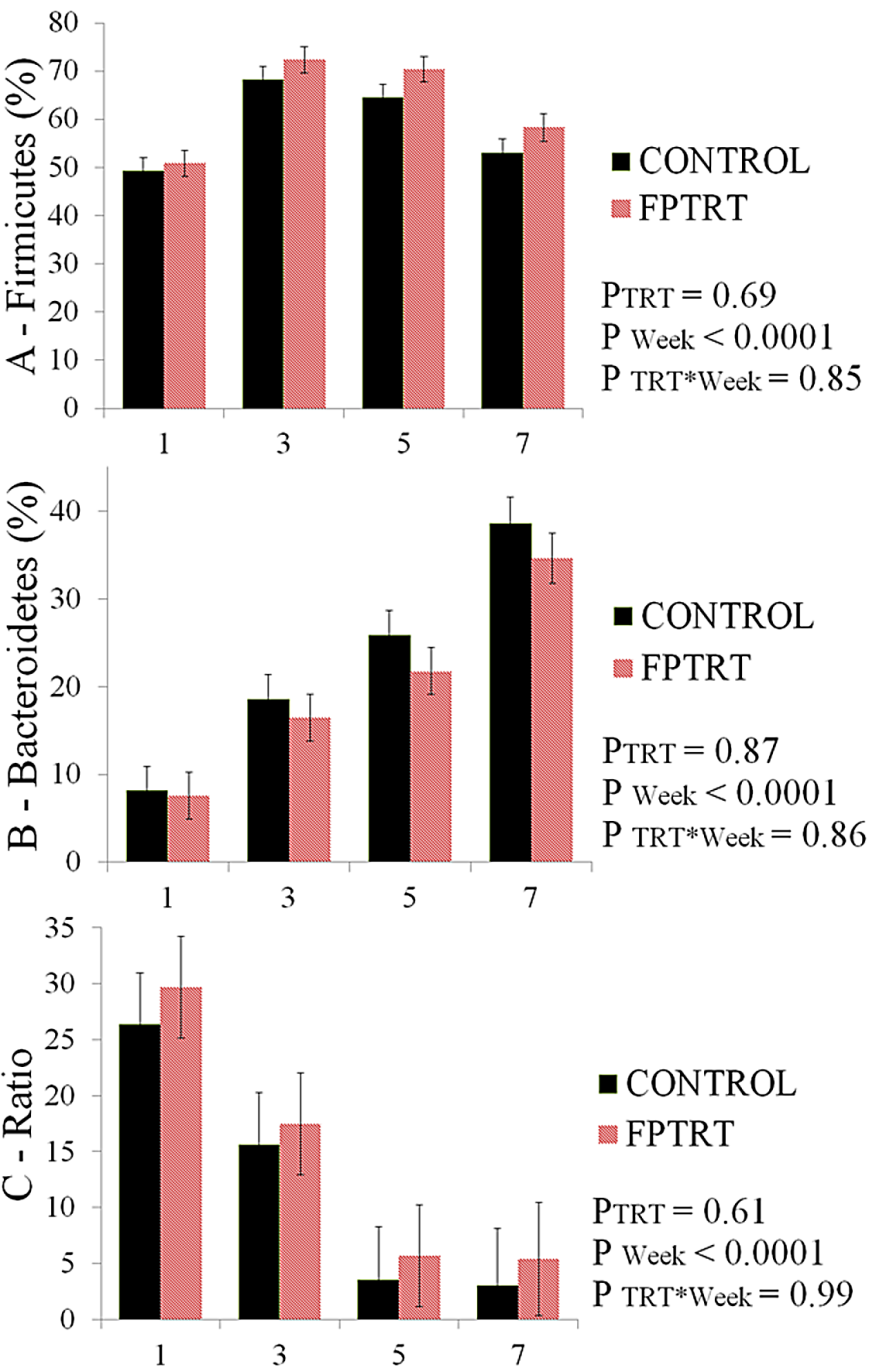
Mean relative abundance of *Firmicutes* and *Bacteroidetes* and their ratio. Field trial. *Firmicutes* mean relative abundance of control calves and FPTRT calves (A). *Bacteroidetes* mean relative abundance (B). *Firmicutes* to *Bacteroidetes* ratio (C). The X axis symbolizes the week of life. The Y axis represents the relative ratio (%) for A and B, and the ratio for graph C. The error bars represent standard errors of the means.

*Firmicutes* and *Bacteroidetes* mean relative abundances are presented in Fig 8A and 8B. The ratio of *Firmicutes* to *Bacteroidetes* is depicted in Fig 8C. A higher ratio was observed in the 1^st^ week and it decreased over time for both groups (*P <* 0.01). Although the FPTRT group had numerically higher ratios than the control group over the time, the differences between the two treatment groups were not statistically significant.

**Fig 9:**
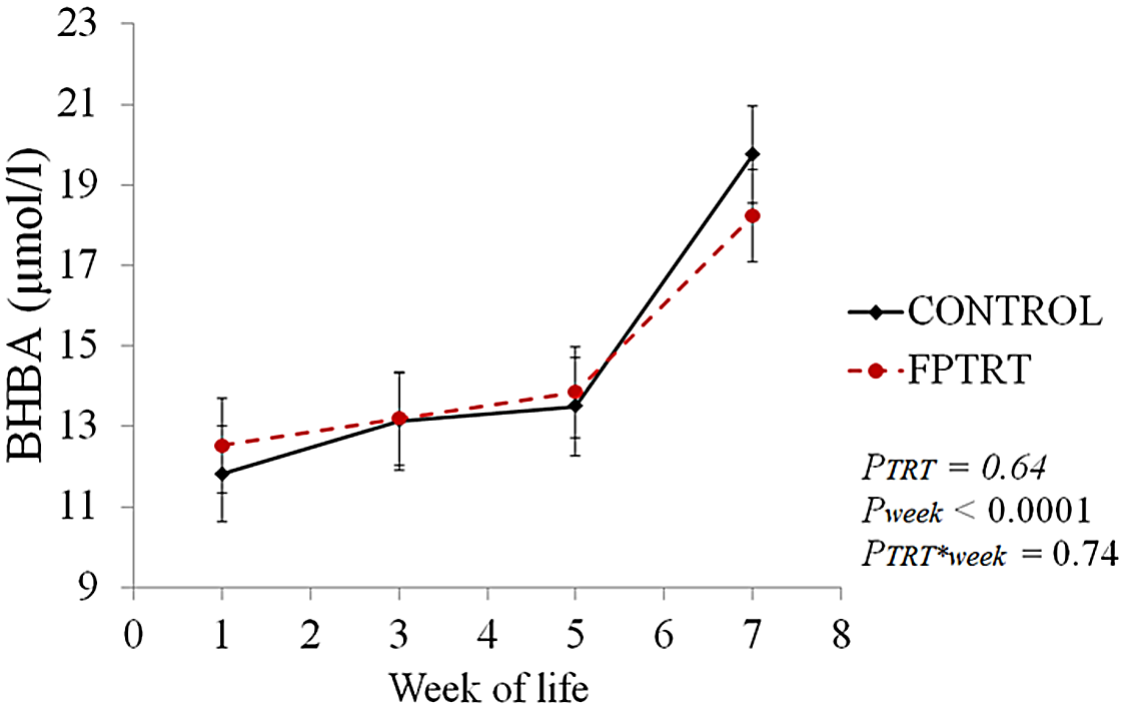
Serum BHBA (μmol/l) concentration over the preweaning period. **Field trial.** Serum BHBA (μmol/l) concentration over the preweaning period by treatment groups: FPTRT (dashed line; oral administration of *F*. *prausnitzii*) or control (solid line; no placebo). The error bars represent standard errors of the means.

Serum BHBA concentration increased significantly over time (*P* < 0.01) and was not affected by treatment (*P* = 0.67), as shown in Fig 9. Overall, the least square means ranged from 11.7 to 19.7 μmol/L.

## Discussion

The use of *F*. *prausnitzii* as a probiotic treatment for newborn dairy calves was assessed. We confirmed its safety and efficacy and moreover determined that oral administration of *F*. *prausnitzii* live culture decreased the incidence of severe diarrhea and related mortality rate, while increasing weight gain in preweaned dairy heifers. This research demonstrates *F*. *prausnitzii* as a novel approach to enhance gastrointestinal health and weight gain in dairy calves.

The anti-inflammatory properties of *F*. *prausnitzii* and the production of butyrate are factors potentially contributing to the positive results observed in treated calves. The anti-inflammatory capacity of *F*. *prausnitzii* and its supernatant has previously been demonstrated both *in vitro* with cultured cells and *in vivo* using TNBS-induced colitis mice models [16,18,20]. Both bacterium and its supernatant induced the production of IL-10, an anti-inflammatory cytokine, while decreasing the secretion of IFN-γ and IL-12, which are pro-inflammatory cytokines [16]. Sokol et al. (2008) also reported that *F*. *prausnitzii* metabolites inhibit the activation of NF-kB and the IL-8 secretion. Additionally, a protective effect of *F*. *prausnitzii* on the intestinal barrier has been suggested [19,20]. We hypothesize that the administration of *F*. *prausnitzii* and its colonization in the lower GIT (cecum and colon), could attenuate the symptoms caused by infectious diarrhea in young calves or possibly prevent new infections from arising. Calf diarrhea plays a key role in the preweaned period. Reducing scours incidence could positively affect animal health and welfare, decrease labor, cost of raising heifers and antibiotic use.

As already stated, butyrate has several known beneficial effects in the intestines. It is a major energy source to colonocytes, it stimulates cell proliferation, differentiation and maturation, and improves colonic barrier function [33,34]. In ruminants, butyrate has been associated with rumen development, having a mitotic effect on ruminal papillae [35], gut maturation [36] and increased weight gain in calves [37]. Considering this, the ability of *F*. *prausnitzii* to produce butyrate could contribute to positive effects observed during colonization of *F*. *prausnitzii* in the large intestines.

Gastrointestinal epithelial cells use a large amount of the energy and nutrients provided by the diet and the microbial fermentation for absorption, transport of nutrients, and tissue maintenance [38]. Dysbiosis and inflammation increase the energy expended by the enterocytes and also impair the intestinal barrier function, increasing the permeability to toxic substances (leaky gut) [39–41]. Therefore, the enhanced growth observed in calves with higher *F*. *prausnitzii* prevalence could be partially explained by the reduced inflammation (decreased secretion of pro-inflammatory cytokines and increased production of anti-inflammatory cytokines) and improved integrity of the intestinal barrier. Healthier calves could potentially have an increased appetite or have the same feed intake but extra energy available for weight gain due to less energy required for maintenance of a healthier gut.

A higher ratio of *Firmicutes* to *Bacteroidetes* has been correlated with obesity in mice and humans [42,43]. Although this ratio was not significantly different between our study groups, it was higher for FPTRT calves compared to control calves. *F*. *prausnitzii* belongs to the phylum *Firmicutes* and has a higher capacity for producing energy than other commensal bacteria. Balamurugan et al. (2010) compared the fecal microbiota of obese and non-obese Indian children using real-time PCR and found significantly higher levels of *F*. *prausnitzii* in the feces of the obese. Oikonomou et al. (2013) was the first to associate greater abundance of *F*. *prausnitzii* with higher weight gain and lower incidence of diarrhea in preweaned calves. Similarly, its relative abundance was increased in FPTRT calves, which gained more weight than controls. Obesity should be avoided in humans and animals, since it can lead to several health disorders (i.e. diabetes mellitus and systemic hypertension). However, increased growth rate is desirable in young production animals. As an example, preweaned dairy heifers are expected to yield more milk in their 1^st^ lactation when they are purposely fed to exceed the nutrient requirements and have a greater ADG [44,45]. Hence, the administration of *F*. *prausnitzii* can be considered as a natural way to improve performance in preweaned heifers.

Calves that presented a high prevalence of *F*. *prausnitzii* on their 1^st^ week of life (HighFP) and that received the additional *F*. *prausnitzii* culture gained significantly more weight compared to HighFP control calves. The *F*. *prausnitzii* isolates used for the probiotic cocktail were selected based on their high production of butyrate, better growth performance and resistance to low pH and bile salts. Treated calves were potentially colonized by more beneficial isolates present in the probiotic cocktail, irrespective of the higher relative abundance of the *F*. *prausnitzii* in their fecal microbiota. Another hypothesis is that HighFP have a gut environment more prone to the colonization of these new isolates administered.

The incidence of severe diarrhea was lower in the FPTRT group, as demonstrated through the survival analysis. Conversely, the mean fecal consistency score was not significantly different between FPTRT calves and control calves. Fecal consistency score alone cannot be a reliable indicator of disease, and diarrhea can be nutritional or infectious. We hypothesize that FPTRT calves were having loose feces due to a large consumption of milk. We do not have milk consumption data in the current study; however, this was similarly reported by Hill et al., 2010, in a study comparing different milk replacer programs. They observed greater average fecal consistency score from calves receiving a higher dry matter amount per day [46].

Short chain fatty acids, mainly butyrate, are converted to BHBA in the rumen epithelium and are metabolized in the liver [47,48]. We first speculated that calves in the FPTRT group would have higher prevalence of *F*. *prausnitzii* and hence have more butyrate, leading to greater serum BHBA concentration than control calves. However, if there was a higher production of butyrate by the *F*. *prausnitzii* in the FPTRT group, it would be in the large intestines [49], where most of the butyrate produced by the microbiota is consumed by the enterocytes and by other bacterial species. This might be one of the reasons that we did not detect differences in the serum BHBA concentration between the two groups. The rise on the BHBA concentration with age reported here is known to be correlated with the increase in starter intake and greater production of VFA by the rumen [24,50,51].

A limited number of longitudinal studies have focused on weekly changes in fecal microbial diversity in calves during the preweaning period [10,52]. In the field trial, *Firmicutes* was the most prevalent phylum, followed by *Bacteroidetes* and *Proteobacteria*. Our results are in agreement with Oikonomou et al. (2013) that studied weekly changes in the fecal microbiota of Holstein heifers. Klein-Jöbstl et al. (2014) collected fecal samples from six Simmental calves across six time points ranging from birth until one week after weaning [52]. Calves were housed individually and were fed saleable pasteurized whole milk. They reported the same three phyla as the most prevalent, *Firmicutes*, *Bacteroidetes* and *Proteobacteria*, but in different ratios. *Firmicutes* was also the major phylum present in the in the fecal microbiota of one to four week old dairy calves fed non-pasteurized waste milk [53]. This cross-sectional study collected samples from 15 calves from each age group, which does not account for the individual variation over time. In addition, the increase in the Shannon index with age observed in the field trial is in agreement with the three studies cited above [10,52,53] and demonstrate that the calves’ gut microbiota was increasing in diversity over time due to the consumption of solid feed.

The use of sterile cotton tipped swabs to collect fecal samples from the rectum requires less handling of animals and permits sampling of microbes encountered in the rectal mucosa and in the fecal material. A study by Malmuthuge et al. (2014) evaluated the taxonomic discrimination of commensal bacteria among the gastrointestinal tract (GIT) of preweaned calves by collecting tissue and content samples from the rumen, jejunum, ileum, cecum, and colon from 8 calves. The amplified 16S rRNA products were pooled by GIT segment and sequenced. They observed that *F*. *prausnitzii* was predominant in the mucosa-associated community from the cecum and colon of preweaned calves’ samples. Therefore, we considered the rectal swab as a better method for fecal sample collection, especially for targeting *F*. *prausnitzii*.

In conclusion, our studies demonstrated that *F*. *prausnitzii* administration to newborn calves is safe and proved the concept that this commensal bacterium is a promising probiotic for newborn calves. Further research is needed to evaluate *in vivo* mechanisms of action and interactions between this microbe, the gut microbiota and the host.

## Supporting Information

S1 Fig Distribution of scores of fecal consistency. Safety trial. Distribution of scores of fecal consistency (0 = well-formed; 1 = semi-formed; 2 = loose or watery feces not containing blood; and 3 = loose or watery feces containing blood) during the 14 days of life of the calves in the safety trial by treatment group.
(TIFF)

S2 Fig Distribution of scores of dehydration. Safety trial. Distribution of scores of dehydration (0 = euhydrated; 1 = skin tented 2 to 6s; 2 = skin tented 6 to 10s; and 3 = skin tented ≥ 10s) during the 14 days of life of the calves in the safety trial by treatment group.
(TIFF)

S3 Fig Distribution of scores of attitude. Safety trial. Distribution of scores of attitude (0 = alert; 1 = depressed; and 2 = non responsive) during the 14 days of life of the calves in the safety trial by treatment group.
(TIFF)

S4 Fig Distribution of scores of appetite. Safety trial. Distribution of scores of appetite (0 = normal; 1 = consumed ½ bottle; 2 = consumed 1/4 bottle; and 3 = forced fed) during the 14 days of life of the calves in the safety trial by treatment group.
(TIFF)
