# Expression of Concern: Oral Administration of *Faecalibacterium prausnitzii* Decreased the Incidence of Severe Diarrhea and Related Mortality Rate and Increased Weight Gain in Preweaned Dairy Heifers

**DOI:** 10.1371/journal.pone.0280713

**Published:** 2023-01-17

**Authors:** The PLOS ONE Editors

After publication of this article [1], concerns were raised about:

- The lack of original data files supporting this article’s [1] results;
- Differences between results reported in the first author’s thesis [2] and this article [1];
- The potential effects of sodium bicarbonate not being addressed through a control group;
- Whether all potentially competing interests were disclosed in full.

Contrary to the declaration in the Data Availability Statement, and in violation of PLOS’ Data Availability Policy, the original data files supporting the article’s results were not provided with the article. The first and corresponding authors indicated that the original raw data supporting results reported in this article [1] are no longer available.

In response to the remaining concerns, the first author claimed that the differences between their thesis [2] and this article [1] reflect changes made during the article’s peer review and revision process. However, without the primary data, this cannot be fully resolved. The first author also acknowledged that ideally there would have been a sodium bicarbonate-only group, and stated they were unable to include this group due to research constraints. Based on this aspect of the study design it is not possible to clarify whether the sodium bicarbonate treatment affected any of the study outcomes.

The article’s Competing Interests statement is updated to: “The authors have read the journal’s policy and have the following competing interests to declare: This study was funded by Merck Animal Health. RCB is the inventor of the patents US9700586B2: Probiotic compositions and methods (filed 17/02/13, published 11/07/17), WO2013130624: Probiotic compositions and methods (filed 27/02/13, published, 06/09/13) and US20150044172A1: Probiotic compositions and methods (filed 27/02/13, published 12/02/15). RCB is also the founder, CEO, and President of FERA Animal Health, now FERA Diagnostics and Biologicals. This does not alter our adherence to PLOS ONE policies on sharing data and materials. There are no other patents, products in development or marketed products associated with this research to declare.” The corresponding author stated that FERA’s activities are not related to probiotics or gut health.

In light of the original underlying data no longer being available, this article [1] does not fully comply with PLOS’ Data Availability Policy, and the differences between the thesis [2] and this article [1] cannot be fully resolved. The *PLOS ONE* Editors therefore issue this Expression of Concern.
