# Supplementary figures and images for: Oral Administration of Faecalibacterium prausnitzii Decreased the Incidence of Severe Diarrhea and Related Mortality Rate and Increased Weight Gain in Preweaned Dairy Heifers
Source: PLoS One. 2015 Dec 28;10(12):e0145485. doi: 10.1371/journal.pone.0145485 (PMC4692552; doi:10.1371/journal.pone.0145485)

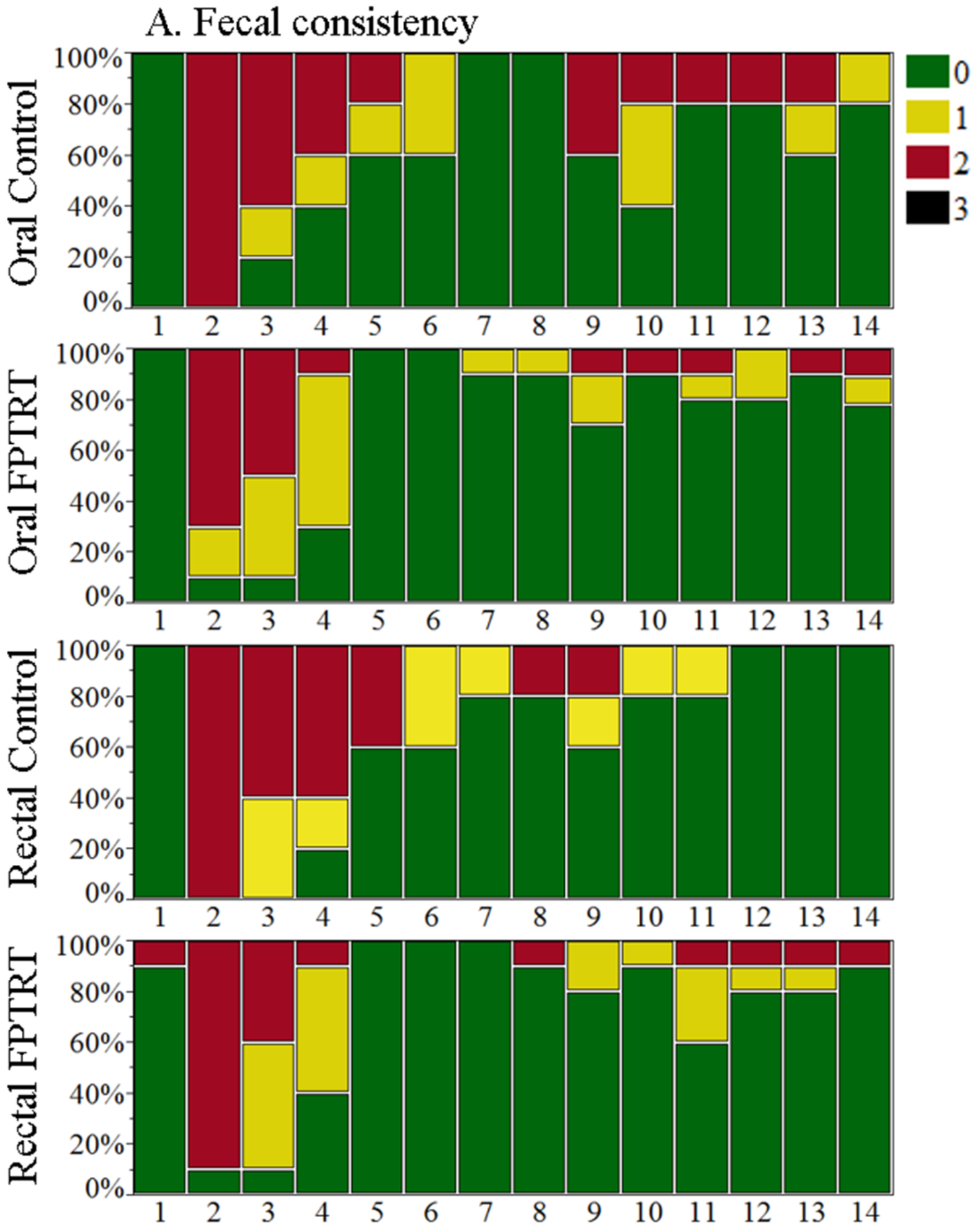

Supplement: S1 Fig — Safety trial. Distribution of scores of fecal consistency (0 = well-formed; 1 = semi-formed; 2 = loose or watery feces not containing blood; and 3 = loose or watery feces containing blood) during the 14 days of life of the calves in the safety trial by treatment group. (TIFF) [file pone.0145485.s001.tiff]

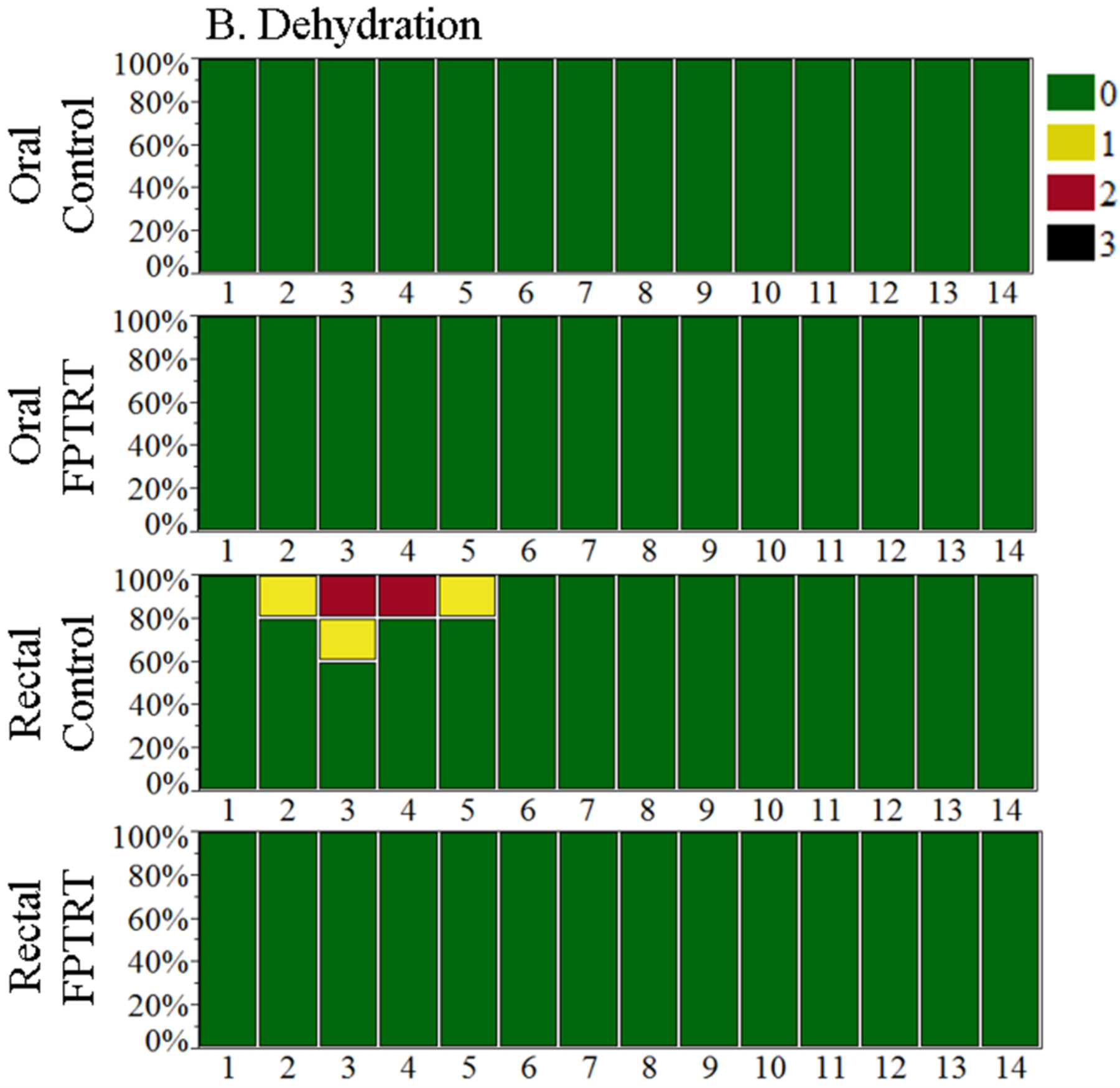

Supplement: S2 Fig — Safety trial. Distribution of scores of dehydration (0 = euhydrated; 1 = skin tented 2 to 6s; 2 = skin tented 6 to 10s; and 3 = skin tented ≥ 10s) during the 14 days of life of the calves in the safety trial by treatment group. (TIFF) [file pone.0145485.s002.tiff]

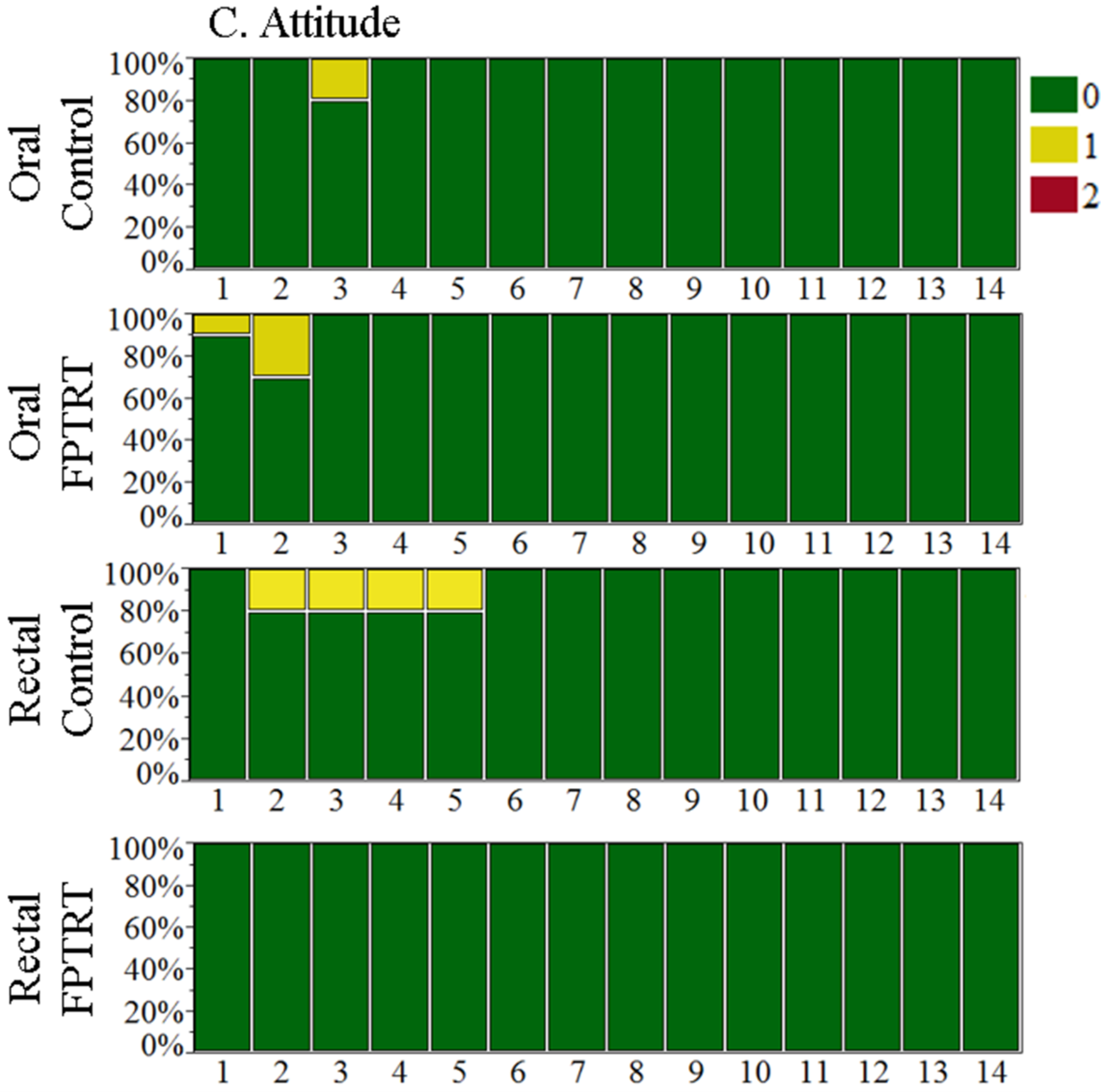

Supplement: S3 Fig — Safety trial. Distribution of scores of attitude (0 = alert; 1 = depressed; and 2 = non responsive) during the 14 days of life of the calves in the safety trial by treatment group. (TIFF) [file pone.0145485.s003.tiff]

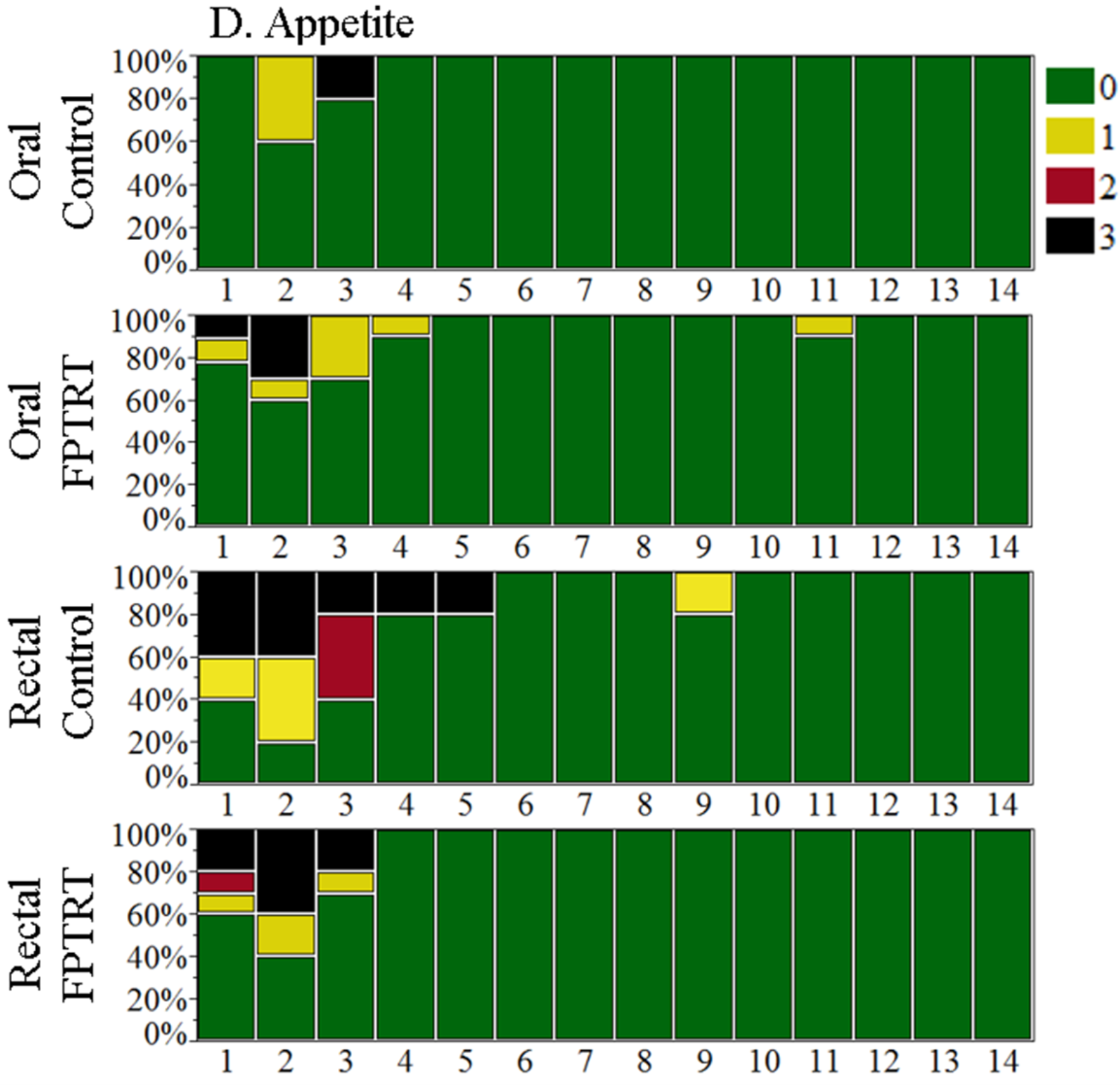

Supplement: S4 Fig — Safety trial. Distribution of scores of appetite (0 = normal; 1 = consumed ½ bottle; 2 = consumed 1/4 bottle; and 3 = forced fed) during the 14 days of life of the calves in the safety trial by treatment group. (TIFF) [file pone.0145485.s004.tiff]
